# Systemic nocardiosis in a dog caused by *Nocardia cyriacigeorgica*

**DOI:** 10.1186/s12917-017-0945-3

**Published:** 2017-01-21

**Authors:** Yesari Eroksuz, Nafia Canan Gursoy, Tolga Karapinar, Burak Karabulut, Canan Akdeniz Incili, Zeynep Yerlikaya, Zulal Asci Toraman, Mehmet Ozkan Timurkan, Hatice Eroksuz

**Affiliations:** 1 Department of Pathology, School of Veterinary Medicine, Firat University, 23 200 Elazig, Turkey; 2 Department of Medical Microbiology, School of Medicine, Inonu University, Malatya, Turkey; 3 Department of Internal Medicine, School of Veterinary Medicine, Firat University, Elazig, Turkey; 4 Department of Microbiology, School of Veterinary Medicine, Firat University, Elazig, Turkey; 5 Department of Medical Microbiology, School of Medicine, Firat University, Elazig, Turkey; 6 Department of Virology, School of Veterinary Medicine, Ataturk University, Erzurum, Turkey

**Keywords:** Canine nocardiosis, *Nocardia cyriacigeorgica*, Systemic involvement

## Abstract

**Background:**

Systemic nocardiosis due to *Nocardia cyriacigeorgica* has not been reported in dogs.

**Case presentation:**

Light and electron microscopy, microbiological culture and molecular identification (PCR) were used to diagnose systemic nocardiosis caused by *Nocardia cyriacigeorgica* in a 3-month-old husky dog. The postmortem changes included multifocal to coalescing, sharply circumscribed pyogranulomatous inflammation and abscess formation in lungs, liver, myocardium, spleen, kidneys, brain, and hilar lymph nodes. The organism was isolated and sequencing of its 16S rRNA allowed its identification and speciation. Examination of the bacterial culture by scanning electron-microscope showed filamentous branching with fragmentation into widely bacillary and cocoid forms of the bacteria. There was no history of immunosupressive drug administration and infection by the immunosuppresive viral pathogens, canine distemper and parvovirus were excluded via PCR.

**Conclusion:**

*N. cyriacigeorgica* should be considered potential cause of systemic pyogranulomatous lesions in dogs. It is the first reported case of systemic nocardiosis due to *N. cyriacigeorgica* in a dog.

## Background

The genus *Nocardia* comprises a group of obligately aeorobic, weakly or variable gram positive, branching often beaded, flamentous bacteria belonging to the family *Nocardiaceae*, together with the genus *Rhodococcus*, within the suborder *Corynebacterineae* of the *Actinobacteria* [1, 2]. *Nocardiae* are nonmotile, facultatively intracellular pathogen in humans and, domestic and wild animals. Furthermore, they cause significant infections in cats, horses, goat, sheep, pigs, fish, birds, marine mammals and a variety of wild and zoo animals [1–3]. The other and separate form of disease is the bovine mastitis occuring in dairy cattle and also it progresses into systemic infection [1]. The microorganisms are ubiquitously present worldwide in the environment including soil, water, or plant material [4].

Molecular methods allowed the categorization of the *N. asteroides* complex into defined antimicrobial-susceptibility patterns [4]. As a result of greater diagnostic capability, following reports in humans indicated that *N. cyriacigeorgica* infections occur worldwide distribution including in Turkey [5–7]. Nocardial infections in dogs result from inhalation, ingestion or subcutaneous transmission characterized by acute or chronic, pyogranulomatous to suppurative inflammatory reactions either in localized organs (skin or lungs) or disseminated to more than one organ. Most of the *Nocardia* isolates in dogs are *Nocardia asteroides complex (*which contains multiple subspecies) [1]. In animals; *N. cyriacigeorgica* infection has been reported in a cat with vertebral osteomyelitis [8]. Furthermore, it was demonstrated that *N. cyriacigeorgica* is a potentially pathogen for mice [9]. However, to the authors’ knowledge, there is no report of *N. cyriacigeorgica* infection in dogs. The present report describes pathological, ultrastructural, microbiological and molecular findings of nocardiosis in a 12-week-old, intact female husky dog caused by *N. cyriacigeorgica*.

## Case presentation

A 12-week-old, intact female husky dog which had been brought from Kayseri, another city at 600 km away from Elazig, 4 weeks earlier, presented to the Firat University Veterinary Teaching Hospital for physical examination. Roundworm infection was diagnosed by the fecal flotation and the treatment promptly was started with pyrantel pamoate (7.50 mg/kg, peroral, Kontil®, Husnu Arslan, Turkey). The dog returned to the Firat University Veterinary Teaching Hospital with the chief compliant of anorexia and vomitting 3 days later. Physical examination revealed increased rectal temperature (40.4^0^C), hyperemic mucous membranes, and wheezing during lung auscultation. Pulse and respiratory rates were 200 beats/min and 80 breaths/min, respectively. The dog was given ampicillin-sulbactam (20 mg/kg, twice daily, intramuscular, Ampisid®, Mustafa Nevzat, Turkey) and enrofloxacin (5 mg/kg, once daily, intramuscular, Baytril®-K, Bayer, Germany). Intravenous fluid therapy of lactated ringer with 5.0% dextrose was administered. Despite therapatic interventions, dog’s clinical condition deteriored and convulsions and muscle tremors in a continous manner began 7 days later. Due to poor prognosis, the owners elected euthanasia. The dog was euthanized and postmortem examination was performed immediately. Tissue samples of liver, lungs, spleen, pancreas, brain, small and large intestines, heart, pericardium and kidneys were fixed in 10% neutral formalin, processed routinely, embedded in paraffin and sectioned at 4-5 μm and stained with hematoxylin-eosine (H&E). Selected tissue sections were stained Gomori’s methenamine silver nitrate (GMS), Brown-Brenn method, Periodic acid Schiff-reaction (PAS), Ziehl-Neelsen (ZN) stain, and Fite Faraco (Fites) stain.

**Fig. 1:**
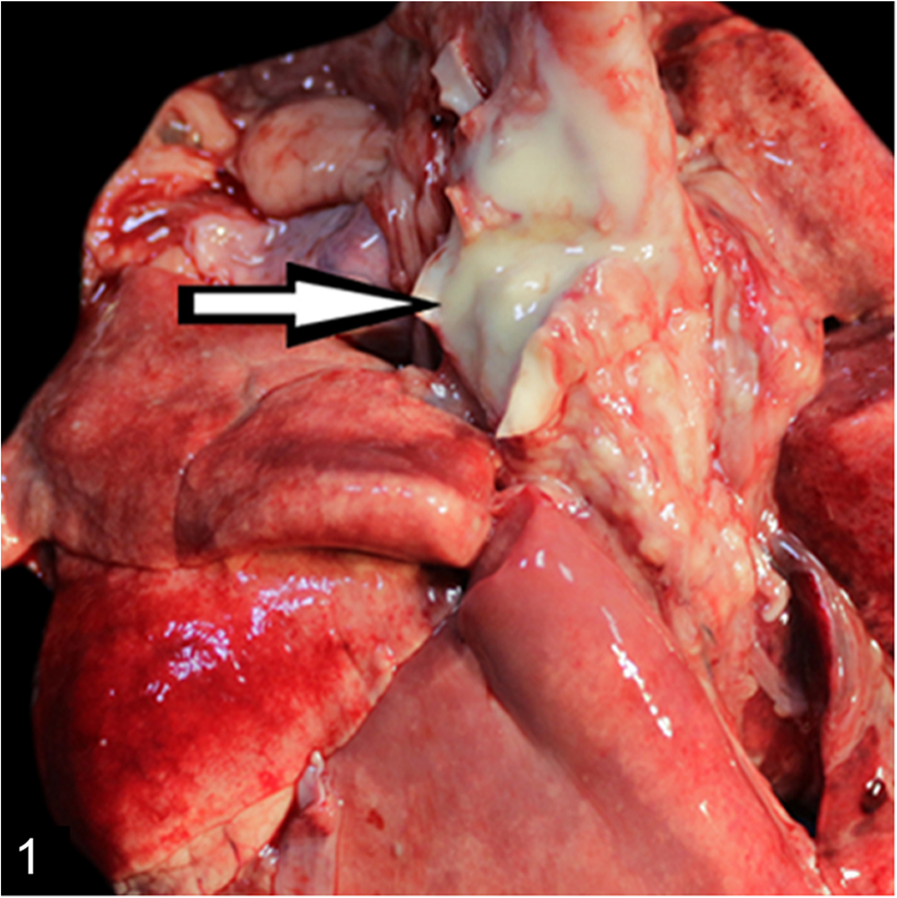
Severe suppurative lymphadenitis in a bronchial lymph node (*arrow*)

**Fig. 2:**
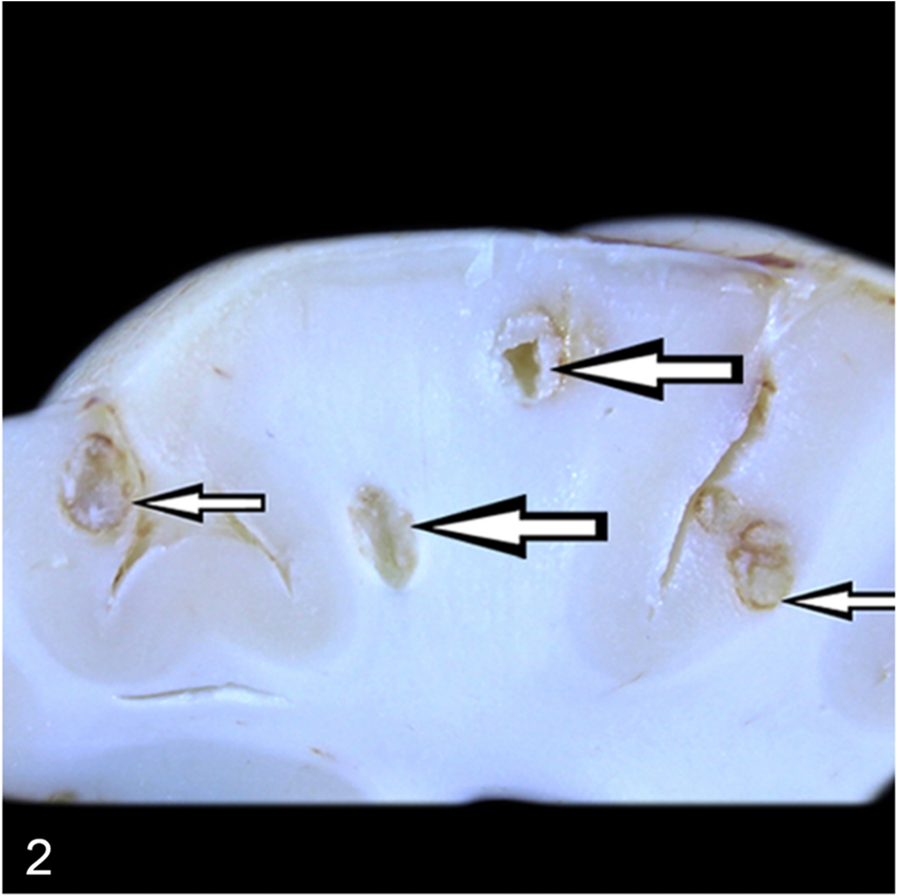
Coronal section of left frontal lobe showing multifocal-coalescing abscesses (*arrows*)

**Fig. 3:**
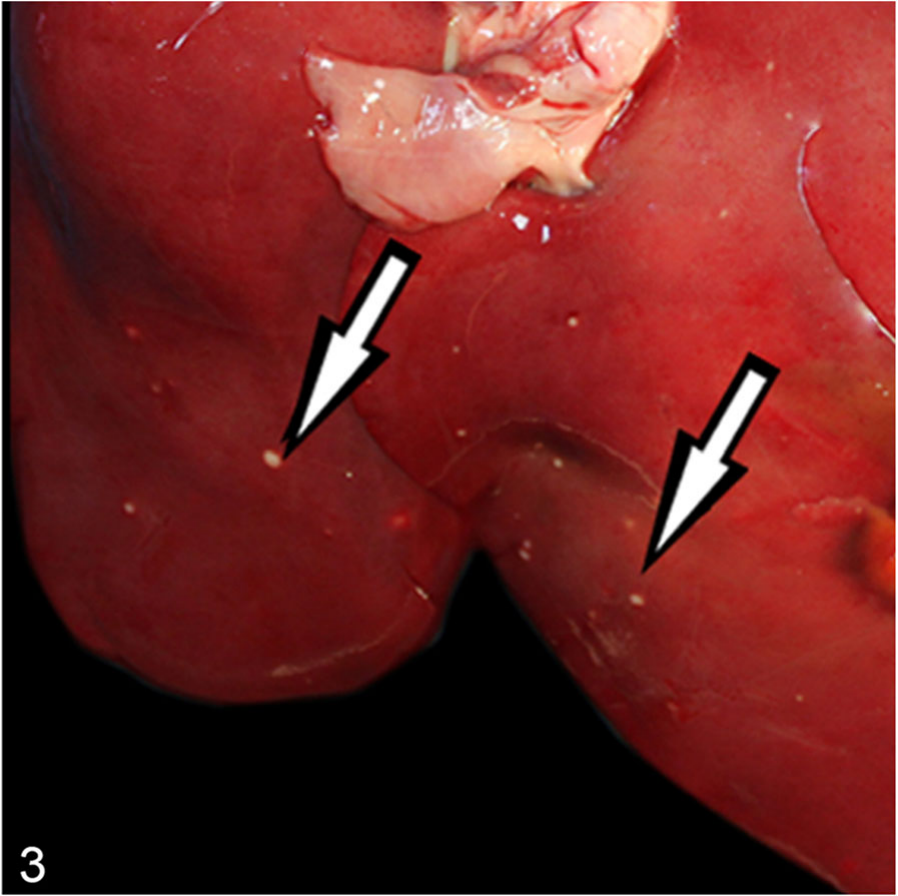
Multifocal, pyogranulomatous hepatitis (*arrows*)

**Fig. 4:**
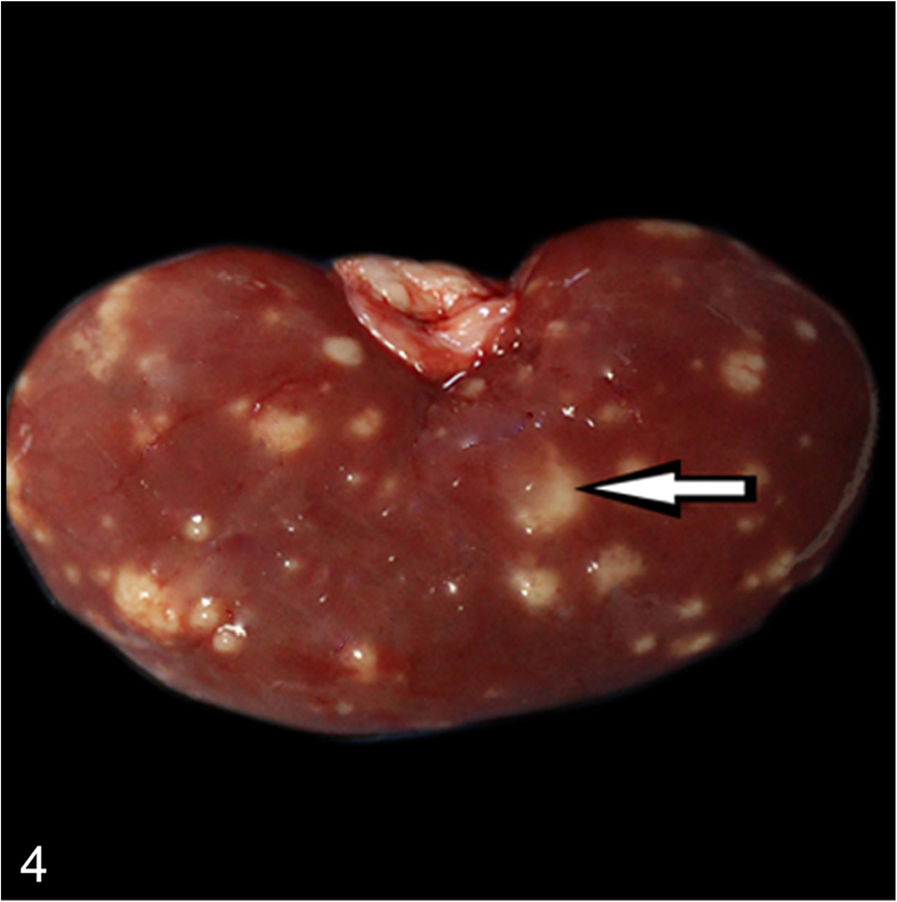
Multifocal, pyogranulomatous nephritis (*arrow*)

At necropsy, the most prominent changes were multifocal to coalescing foci of pyogranulomatous inflammation or abscess formation in liver, lungs, heart muscle, spleen, kidneys, pulmonary lymph nodes and brain (Figs. 1, 2, 3 and 4). Coronal sections of the brain including parietal, frontal, rostral, optic chisam, intermediate hypotalamus, mamillary bodies, oculamator nucleus, rostral nucleus, caudal nucleus, pons and cerebellum showed multifocal-coalescing abscesses that were located at the border of white and gray matter, and deep portions of brain sulci. The meninges were thickened and opaque in appearence. Pulmonary hilar lymph nodes also showed abscessation. The thoracic cavitiy and pericardial sac contained 30 ml and 15 ml fibrino-hemorrhagic exudate, respectively. There were multifocal petechial hemorrhage in epicardial and diaphragmatic surfaces.

**Fig. 5:**
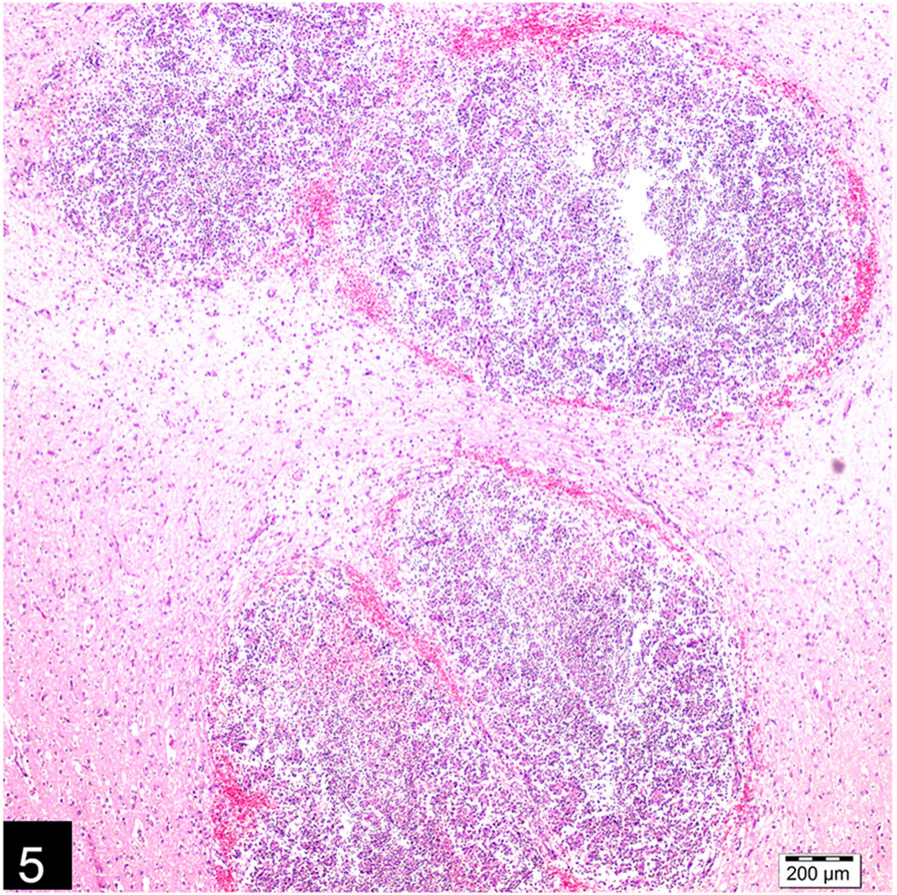
Multifocal, coalescing abscesses in the cerebral cortex, (H&E)

**Fig. 6:**
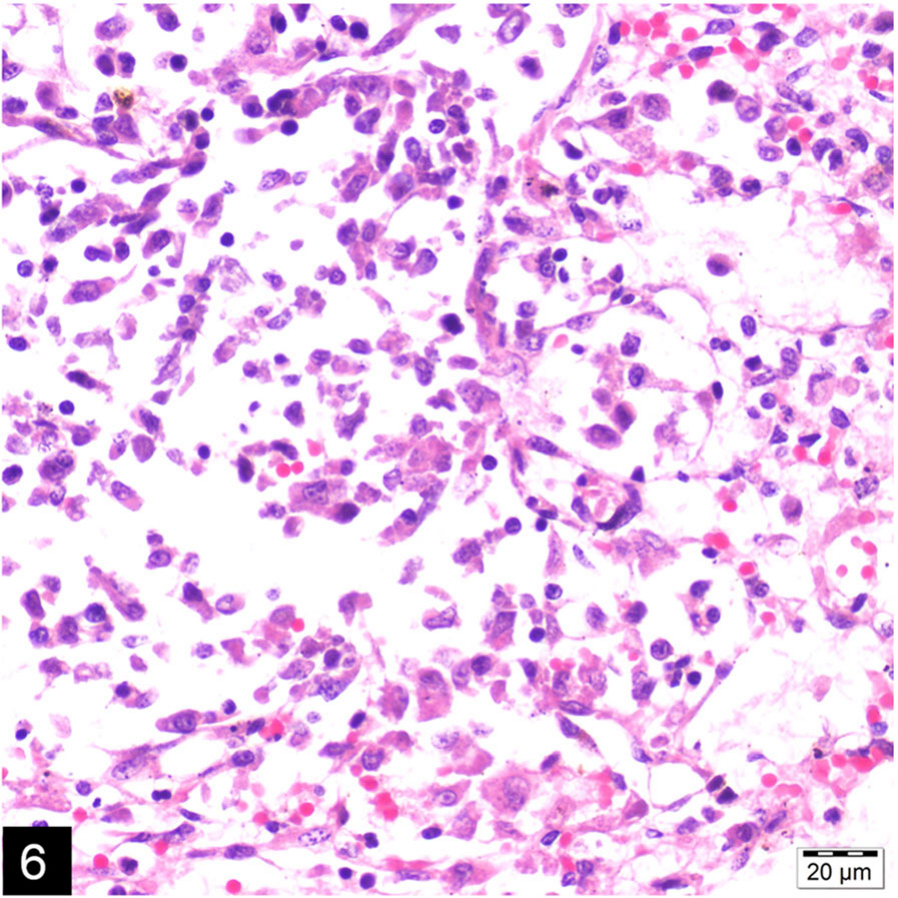
Pyogranuloma containing epithelioid macrophages, neutrophils and lymphocytes in the kidney, (H&E)

**Fig. 7:**
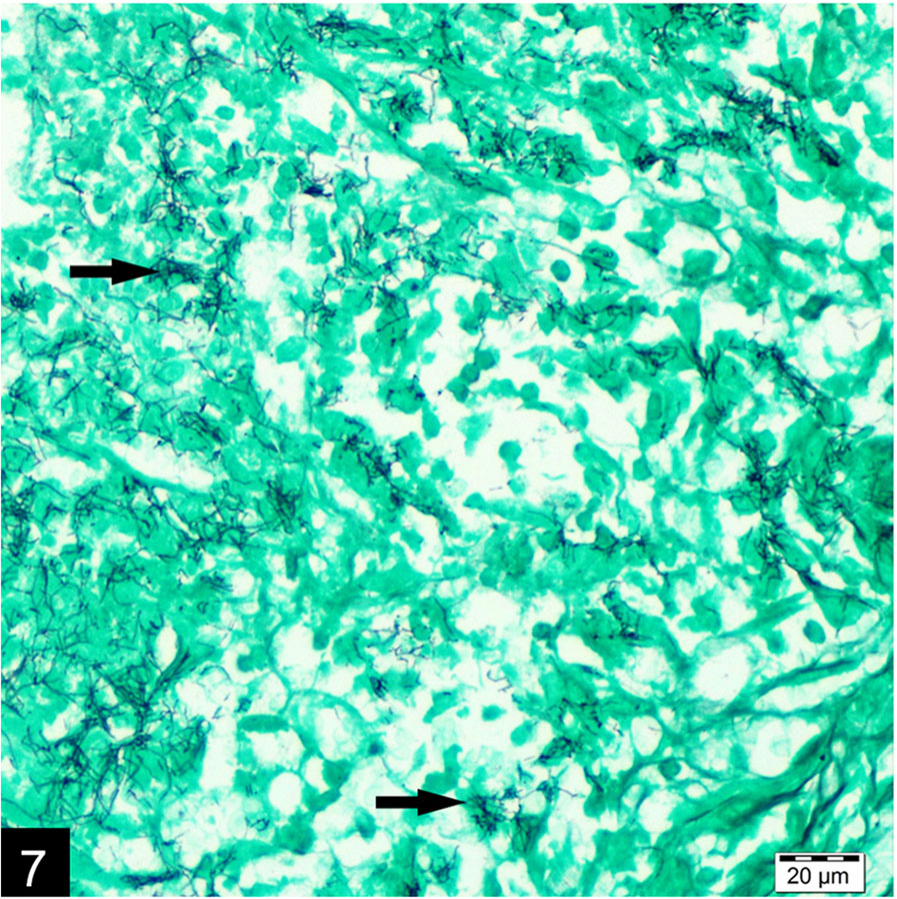
Intralesional, filamentous, branching argyrophilic micro-oganisms (arrows) in the brain, (GMS)

**Fig. 8:**
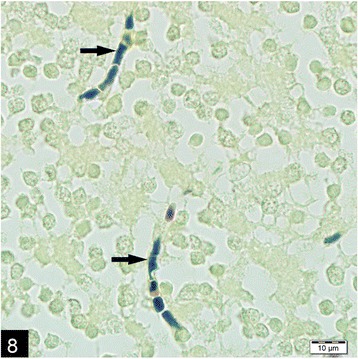
Gram positive, intralesional, beaded micro-organisms (arrows) in the brain, (Brown and Brenn)

Microscopically, the lungs, spleen, heart, liver and kidneys had multifocal to coalescing pyogranulomas (Figs. 5 and 6). These pyogranulomas were located subendocardial myocardium in the heart, renal cortex, subcapsular and cut surfaces of lungs and liver. Larger granulomas with necrotic centers had branching bacterial colonies, degenerated and viable polymorphnuclear leucocytes surrounded by lymphocytes, a peripheral rim of epitheloid macrophages, and scant multi-nucleated giant cells with or without fibrous capsule. Smaller pyogranulomas lacked a central necrosis and contained more polymorphonuclear leucocytes and fewer epitheloid cells. The brain had multifocal to coalescing abscess formations in most examined sections of cerebrum and cerebellum. Additional changes included moderate lymphoplasmacytic and neutrophilic inflammatory reaction in *Virchow-Robin* spaces, and mild to moderate mixed meningitis. Blood vessels surrounding the granulomas had reactive endothelial cells. Bronchial lymph nodes had also varying sized of abscess formation characterized by acumulation of viable and degenerated polymorphnuclear leucocytes and few mononuclear cell infiltration. Intra-lesional bacterial colonies were detected as beaded or breached. These organisms were best visualized by GMS and lesser extend with Gram stain and (Figs. 7 and 8), modified ZN, whereas they gave negative reaction to PAS and ZN. Microorganisms were also present individually with no inflammatory reaction in pulmonary alveolar spaces and cerebral neuropil. Additionally, the lungs showed moderate diffuse alveolar histiocytosis, multifocal pleural fibrosis and pleuritis. Hepatic lesions included sinusoidal congestion, *Kupffer* cell activation and diffuse severe cloudy swelling. There was mild to moderate interstitial edema and congestion of blood vessels within myocardium. Thymus was normal grossly and histopathologically, mesenterial and portal lymph nodes were hyperemic and edematous grossly, and there was lymphoid and histiocytic hyperplasia histologically.

The bacteria isolated from lung tissue and hilar lymph nodes were cultured and identified by morphological characteristics and biochemical tests. Bacteria were grown on blood agar base containing 5.0% sterile defibrinated sheep blood at 37.5 °C for 48 h in aerobic and microaerophilic (10% CO_2_) conditions. Incubation at 45 °C was also used to differentiate *N. asteroides* from other *Nocardia* species. At all of the stated conditions, the bacteria grew as typical chalky-white colonies. The bacteria stained positively with the Gram stain and were filamentous. The catalase reaction was positive while the oxidase reaction was negative. The bacteria were able to ferment mannose, lactose and glucose and were unable to ferment mannitol, galactose and xylose.

**Fig. 9:**
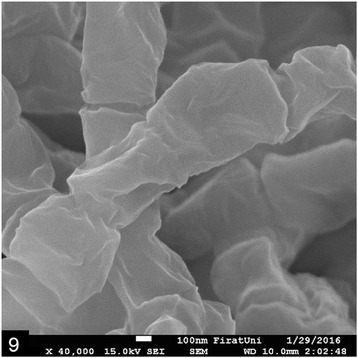
Scanning electron-microscopic (x40.000) appearance of filamentous, beaded microorganisms in bacteriologic culture

Pure colonies of microorganisms obtained from sheep blood agar (5.0%) were re-suspended in 5 mL of NaCl solution (0.85%) in glass tubes. The bacteria were fixed in 2.50% glutaraldehyde in 0.1 M phosphate buffer (pH 7.3) and then post-fixed in 1.0% osmium tetroxide solution in same buffer solution. Finally, they were dehydrated in graded ethanol solution and coated with a gold layer (10 nm). The culture material was analyzed using scanning electron microscopy, which showed details filamentous to coccobacillary organisms of *N. asteroides* measuring 467 ± 85 by 813 ± 120 nm, branching beads (Fig. 9).

To test for canine distemper virus and parvovirus, viral nucleic acid was extracted from paraffin-embedded tissues (brain, liver, mesenterial lymph node) using a Viral Nucleic Acid Extraction Kit (Vivantis®, Malaysia). The follow-up cDNA synthesis was carried out using a first strand cDNA Synthesis Kit (Fermentas®, ThermoFisher Scientific®, USA) as described by the manufacturer’s protocol, using random primers. For diagnosis, primers were chosen for the hemagglutinin gene of canine distemper virus and the VP2 gene for canine parvovirus. PCR was carried out in a total volume 50 μL containing 5 μL of cDNA and 45 μL of master mix (Maxima Hot Start PCR Master Mix (2X), ThermoFisher Scientific, USA). The PCR was initiated by a single denaturation step at 94 °C for 5 min, followed by 35 cycles of 1 min at 94 °C, annealing at 50 °C for 1 min and extension for 1 min at 72 °C and finally 7 min at 72 °C for a final extension. Five microliters of each PCR product were analyzed on 0.5% agarose gel (Prona®, Spain) containing ethidium bromide. Reverse transcriptase polymerase chain reaction (RT-PCR) was used to detect RNA and DNA of canine distemper (CD) and canine parvovirus (CP). All the samples for CD and CP were negative via PCR.

16S rRNA and hps65 PCR and sequence analysis were performed for accurate species identification; partial 16S rRNA gene region was sequenced by using specific primers of p8FPL 5′ AGT TTG ATC ATG GCT CAG-3′ and p806R 5′-GGA CTA CCA GGG TAT CTA AT-3′. Genomic DNA extraction of the isolate grown on blood agar was performed by using QIAsymphony Virus/Pathogen Midi Kit in QIAsymphony SP automatic system. After the isolation, PCR was performed to selectively amplify the 16S rDNA region in a thermal cycler (GeneAmp® PCR System 9700, Applied Biosystems, USA) using the following program; an initial denaturation step of 3 min at 94 °C followed by 35 cycles of 30 s. at 94 °C then 30 s. 60 °C, and ending with a final elongation step for 1 min at 72 °C. PCR products (~800 bp) were electrophoresed in 1.50% agarose gel with ethidium bromide and photographed under UV illumination and were directly purified with a PCR purification kit (QIAquick®, Qiagen, Netherlands) according to the manufacturer’s recommendations. A total of 35 cycles of dideoxynucleotide sequencing procedure was performed using dye terminator kit (ABI Prism BigDye® Terminator v3.1 kit, Applied Biosystems, USA) in both directions at 96 °C/10 s, 50 °C/5 s and 60 °C/6 min. Sequence products were uploaded to Genetic Analyser (ABI Prism® 310 Genetic Analizer, USA) and the chromatograms were compared with the isolates in Gen Bank using BLAST (Basic Local Alignment Tool) program. Sequences of the fragment yielded 99% similarity with *N. cyriacigeorgica* reference sequence with E-value 0 (GenBank accession no: KU356894). The 16S rRNA gene showed 99% identity with the sequences of other *N. cyriacigeorgic*a strains obtained from GenBank. In vitro antimicrobial susceptibility test using disc diffusion method [10] indicated that the isolate is susceptible to ampicillin sulbactam, amoxicillin clavulanic acid, cefepime, imipenem, gentamicine, tigecycline and linezolid.

## Discussion

Nocardial infections in dogs are serious and a review of literature in 1983 indicated that 52 of 46 of dogs (88.5%) died or were euthanized despite the therapy given [1]. The lung is generally considered as primary site of involvement in canine nocardiosis. For the present case, since there was no tonsillar or submandibular lymph node nor skin involvement, respiratory system seems to be primary site of involvement and disseminated to other organs or systems. This was explained by nocardial lesions in lungs or in a particular organ eroding into vessels and invading other organs [1, 2].

## Conclusion

The clinical presentation of nocardial infections in dogs was reportedly very similar to that seen in humans and proposed as a good model for human nocardiosis [2], however, unlike to humans [1, 2, 5, 6, 11], the infection appears to be primary in most instance without underlying cause in dogs. Of 52 canine Nocardia spp. cases in veterinary literature by 1983, no predisposing factor was identifiable in 35 (63.70%) of the dogs, and canine distemper was diagnosed 4 dogs (corresponding 7.70%) [2]. Of 831 N. asteroides infection in human medical literature by 1994, 265 had systemic infections and half of these infections caused also lesions in central nervous system [1]. The age of present case is consistent with the literature implying that Nocardia is primary pathogen for the young dog; hence 34 of 52 dogs, 65.40% were less than 1 year of age [2]. Similarly, juvenile marine mammals were reportedly affected more than the adult animals [3]. The primary differential diagnosis for the present case include Toxoplasma gondii, fungal and mycobacterial infections. Larger populations should be studied to improve our understanding of the characteristics of systemic N. cyriacigeorgica infection in dogs, however N. cyriacigeorgica infection should be considered as differential diagnosis in dogs with systemic pyogranulomatous lesions.

## Abbreviations

PCR: Polimerase chain reaction
GMS: Gomori’s methenamine silver nitrate staining method
H&E: Hematoxylin and eosine staining method
PAS: Periodic acid Schiff-reaction staining method
ZN: Ziehl-Neelsen staining method
CD: Canine distemper
CP: Canine parvovirus
UV: Ultraviolet
